# Mesothelial cyst of uterus in a nullipara patient: A case report

**DOI:** 10.1097/MD.0000000000033159

**Published:** 2023-03-03

**Authors:** Xiu-Cong Ren, Wei Liu, Li-Rong Hu, Min Mao

**Affiliations:** a Department of Gynecology, Chengdu First People’s Hospital, Chengdu, Sichuan Province, China; b Department of Pathology, Chengdu First People’s Hospital, Chengdu, Sichuan Province, China; c Department of Ultrasound, Chengdu First People’s Hospital, Chengdu, Sichuan Province, China.

**Keywords:** immunohistochemistry, laparoscopic uterine cystectomy, supersonic examination, uterine mesothelial cyst

## Abstract

Uterine mesothelial cysts represent a diagnostic challenge because of their low incidence, with very few cases reported in the English literature.

**Patient concerns:**

We report the case of a 27-year-old nullipara woman complaining of self-discovery of a mass in the abdomen for 1 week. Supersonic examination revealed a pelvic cystic lesion measuring 8.9 × 8.2 cm. The patient underwent exploratory single-port laparoscopic surgery and had a large uterine cystic mass located within the posterior wall of the uterus.

**Diagnosis:**

After excision of the uterine cyst, the final histopathological diagnosis was uterine mesothelial cyst.

**Interventions:**

We treated her with a single-port laparoscopic uterine cystectomy.

**Outcomes:**

Close follow-up of the case for 2 years showed that the patient was free of any symptoms, and no recurrence was noted.

**Lessons:**

Uterine mesothelial cysts are extremely rare. They are often misdiagnosed by clinicians as extrauterine masses or cystic degeneration of leiomyomas. This report aims to share a rare case of uterine mesothelial cyst and improve gynecologists’ academic vision of the disease.

## 1. Introduction

In the uterus, cysts are classified as either acquired or congenital. Various acquired uterine cystic lesions have been reported in humans, and are commonly associated with cystic degeneration of leiomyomas, adenomyosis, serosal inclusion cysts, and cystic endometrial hyperplasia.^[1–3]^ Congenital uterine cysts may arise from the paramesonephric (Müllerian) and mesonephric (Wolffian) ducts.^[4]^ Herein, we present the case of a woman who underwent surgery with a preoperative diagnosis of an extrauterine mass only for postoperative diagnosis of the uterine mesothelial cyst. We also reviewed and summarized the clinical characteristics of reported uterine mesothelial cysts (UMCs) to help clinicians obtain a better understanding of UMCs and improve the diagnosis, treatment, and postoperative recovery of patients.

## 2. Case presentation

**Figure 1.**
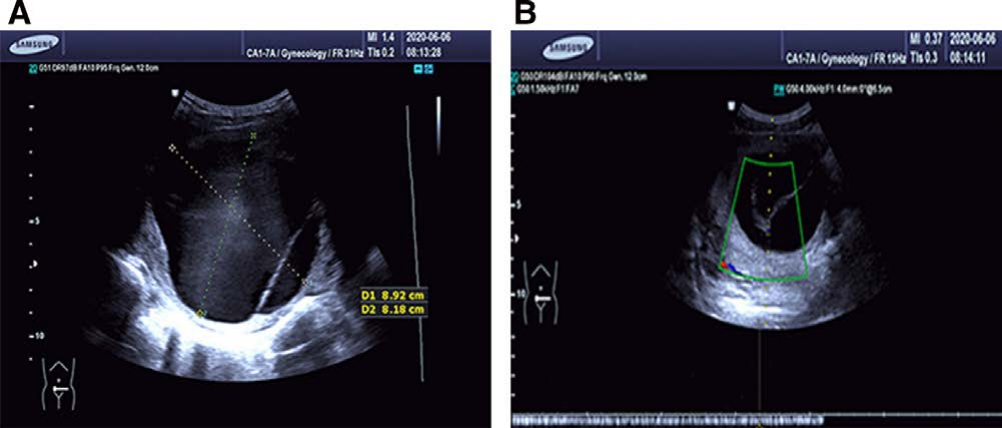
(A) Supersonic examination reveals a pelvic cystic lesion measuring 8.9 × 8.2 cm. (B) Supersonic examination shows the septa within the pelvic cystic lesion.

**Figure 2.**
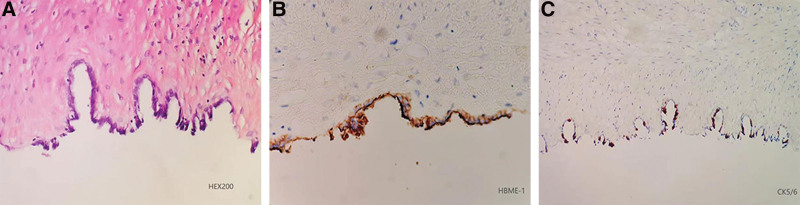
Histopathology and histochemical staining of the uterine cyst. (A) The cyst lined by a single layer of flattened cuboidal to columnar epithelium (200×); (B) Immunohistochemical staining of human bone marrow endothelial marker-1 (HBME-1) (+) (200×); (C) Immunohistochemical staining of cytokeratin 5/6 (+) (100 ×). HBME-1 = human bone marrow endothelial marker-1.

A 27-year-old nullipara woman with a complaint of self-discovery of a mass in the abdomen for 1 week was admitted to Chengdu First People’s Hospital on June 16, 2020. The patient had no relevant medical history. In addition, she had never had sexual intercourse and had no abdominal pain, abnormal uterine bleeding, or dysmenorrhea. Clinically, the abdomen was soft and non-tender, with a palpable mass measuring approximately 10 × 9 cm. Transabdominal ultrasound showed a pelvic anomaly consisting of a multiloculated cyst measuring 8.9 × 8.2 cm, and no solid component was seen (Fig. 1). The tumor marker levels were within the normal range, and the results of laboratory examinations were normal. An erroneous diagnosis of an extrauterine mass was made preoperatively. An exploratory single-port laparoscopic surgery was performed. During the operation, a uterine cyst was observed in the posterior wall of the uterine body and contained clear light-yellow fluid. No other abnormalities were observed. The operation was performed under general anesthesia by a senior skilled surgeon using the stripping technique, who was particularly aware of the need to avoid damaging the healthy part of the uterus. The uterine cyst was plurilocular and adjacent to the myometrium of the uterus and was not associated with the uterine cavity. The cyst wall was smooth and without a wall nodule. It was not easily excised, and sharp dissection was required because the boundary between the cyst wall and myometrium was not clear. The uterine cyst was removed to the extent possible. Laparoscopic excision of the uterine cyst was performed without complications, and the patient was discharged within 72 hours after surgery. Routine histological analysis and immunohistochemistry were used to exclude malignancy and confirm the diagnosis of uterine cysts for the final categorization. Microscopically, the cyst was lined with a single layer of flattened cuboidal to columnar epithelial cells without focal cilia. There was no obvious atypia, mitosis, or necrosis. The cyst epithelia stained intensely positive for human bone marrow endothelial marker-1 (HBME-1) and cytokeratin 5/6 (Fig. 2), and negative for paired box protein 8 (PAX8) and GATA binding protein 3 (GATA3). Postoperative pathological analysis revealed the presence of a uterine mesothelial cyst. The patient was followed up every 6 months and checked using transabdominal ultrasound at each visit. The patient was free of any symptoms, and resumption of normal menstrual rhythm was observed within 4 weeks after the operation. There was no evidence of uterine cyst recurrence after 2-year postoperative follow-up.

## 3. Discussion

Mesothelial cysts are benign neoplastic lesions derived from mesothelial cells and mainly involve the pelvic and peritoneal peritoneum. It is more common in the round ligament, mesentery, and omentum majus, and less common in the uterus.^[5]^ At first glance, UMCs are not considered in the differential diagnosis because of their rarity. A search of PubMed entries since 1985 retrieved only 1 case report on a mesothelial cyst in the uterine myometrium. In the reported case,^[6]^ the patient experienced a recurrent uterine mesothelial cyst 2 years after laparoscopic cystectomy and eventually underwent a total hysterectomy and bilateral salpingectomy.

The etiology of mesothelial cysts remains unclear, and preoperative diagnosis is difficult. However, postoperative immunohistochemistry would be helpful in the differential diagnosis. The criteria for the immunohistochemical diagnosis of UMCs are not definitive. Currently, HBME-1, calretinin, thrombomodulin, WT1 gene product, and cytokeratin 5/6 are considered the best antibodies for identifying mesothelial differentiation.^[7,8]^ In addition to positive “‘markers,’” the panel should also include antibodies, which should be negative in mesothelial cells, to differentiate them from other diagnoses. PAX8 and GATA3 have been identified as immune markers that differentiate between paramesonephric and mesonephric neoplasms, respectively.^[9,10]^ In the present case, the uterine cyst appeared to be a developmental anomaly derived from the mesothelial cells. This is supported by the observation that the uterine cyst was lined by a single layer of flattened cuboidal to columnar epithelial cells; cyst epithelia were not associated with the endometrial cavity, glands, or stroma; the cyst walls were located immediately subjacent to or embedded in the outer myometrium; and the cyst epithelia were positive for antibodies for HBME-1 and cytokeratin 5/6 and negative for antibodies for PAX8 and GATA3.

Owing to the large space in the abdominal area, small UMCs in this area have no specific symptoms. After the cyst grows, patients may experience lower abdominal discomfort or a palpable abdominal mass. Symptomatic or progressively enlarging cysts are generally treated surgically. Laparoscopic resection is recommended, if possible. It should be noted that during the operation, complete resection is difficult to be performed because part of the cyst wall is embedded in the outer myometrium. In the present case, UMCs did not recur easily after surgery. Laparoscopic uterine cystectomy is associated with a risk of surgical injury to the healthy myometrium near the cyst capsule. It is not clear whether uterine cystectomy affects uterine abruption during pregnancy. Patients who undergo uterus-sparing resection should be managed even more conservatively during future pregnancies and are candidates for pre-labor cesarean birth. For patients who have contraindications to surgery or who have not completed their reproductive program, laparoscopic excision of the uterine cyst can be delayed, and alternative surgical techniques, such as ultrasound-guided puncture with alcohol injection or ultrasound-guided aspiration of UMCs, could be considered. The positive clinical effects of alternative surgical techniques remain to be elucidated.

## 4. Conclusion

In conclusion, mesothelial cysts are rarely present in the uterus, and a definitive diagnosis is usually made intraoperatively and confirmed histopathologically. Awareness of the existence of this rare lesion would prevent misdiagnosis and mistreatment. Although it is difficult to completely excise the cyst wall during surgery, the prognosis of this condition is good.

## Acknowledgment

We are grateful to the patient for providing informed consent for publication.

## Author contributions

**Data curation:** Wei Liu, Li-Rong Hu.

Formal analysis: Li-Rong Hu.

Investigation: Wei Liu.

Writing – original draft: Xiu-Cong Ren, Min Mao.

Writing – review & editing: Min Mao.
